# Enhanced High-Fructose Corn Syrup Production: Immobilizing *Serratia marcescens* Glucose Isomerase on MOF (Co)-525 Reduces Co^2+^ Dependency in Glucose Isomerization to Fructose

**DOI:** 10.3390/foods13040527

**Published:** 2024-02-08

**Authors:** Xu Geng, Yi Li, Ruizhe Wang, Song Jiang, Yingchao Liang, Tao Li, Chen Li, Jin Tao, Zhengqiang Li

**Affiliations:** 1 Key Laboratory for Molecular Enzymology and Engineering of the Ministry of Education, College of Life Sciences, Jilin University, Changchun 130012, China; gengxu21@mails.jlu.edu.cn (X.G.); 15133892503@163.com (S.J.); taoli@jlu.edu.cn (T.L.); 2 National Engineering Research Center for Corn Deep Processing, Jilin COFCO Biochemical Co., Ltd., Changchun 130033, China; li-yi1@cofco.com (Y.L.); liangyc@cofco.com (Y.L.); 3 State Key Laboratory for Diagnosis and Treatment of Severe Zoonotic Infectious Diseases, Key Laboratory for Zoonosis Research of the Ministry of Education, Institute of Zoonosis, College of Veterinary Medicine, Jilin University, Changchun 130062, China; wangrz1204@163.com (R.W.); lc2018@jlu.edu.cn (C.L.)

**Keywords:** *Serratia marcescens* GI, glucose isomerase, MOF (Co)-525, enzyme immobilization

## Abstract

The escalating demand for processed foods has led to the widespread industrial use of glucose isomerase (GI) for high-fructose corn syrup (HFCS) production. This reliance on GIs necessitates continual Co^2+^ supplementation to sustain high catalytic activity across multiple reaction cycles. In this study, *Serratia marcescens* GI (*Sm*GI) was immobilized onto surfaces of the metal-organic framework (MOF) material MOF (Co)-525 to generate MOF (Co)-525-GI for use in catalyzing glucose isomerization to generate fructose. Examination of MOF (Co)-525-GI structural features using scanning electron microscopy-energy dispersive spectroscopy, Fourier-transform infrared spectroscopy, and ultraviolet spectroscopy revealed no structural changes after *Sm*GI immobilization and the addition of Co^2+^. Notably, MOF (Co)-525-GI exhibited optimal catalytic activity at pH 7.5 and 70 °C, with a maximum reaction rate (*V*_max_) of 37.24 ± 1.91 μM/min and Km value of 46.25 ± 3.03 mM observed. Remarkably, immobilized *Sm*GI exhibited sustained high catalytic activity over multiple cycles without continuous Co^2+^ infusion, retaining its molecular structure and 96.38% of its initial activity after six reaction cycles. These results underscore the potential of MOF (Co)-525-GI to serve as a safer and more efficient immobilized enzyme technology compared to traditional GI-based food-processing technologies.

## 1. Introduction

The global demand for high-fructose corn syrup (HFCS), a widely used sweetener, has surged in recent decades, securing its place as the world’s second most consumed sugar source [1,2]. Remarkably, HFCS, with double the sweetness of sucrose at equivalent caloric levels, holds a significant role in the food, beverage, and baking industries due to its non-crystallizing properties at high concentrations [3,4]. Additionally, the use of HFCS in food processing contributes to reduced tooth decay. The production of HFCS hinges on glucose isomerase (GI), a catalyst converting D-glucose to D-fructose by isomerizing aldose to ketose [5,6]. In the enzymatic hydrolysis of starch for HFCS, the rate-limiting step is the glucose isomerization [7]. The isomerization reaction operates within a thermodynamic equilibrium, and elevated reaction temperatures enhance the conversion rate, underscoring the value of heat-resistant GIs for boosting HFCS yield [8,9].

Currently, two primary methods have been employed to enhance the temperature tolerance of glucose isomerase (GI). One approach involves screening genes to identify GI naturally tolerant to high temperatures. Another method focuses on immobilizing native GI onto solid substrates, thereby bolstering enzyme stability at elevated temperatures. Deng et al. utilized the former method, identifying a thermotolerant GI from *Thermobifida fusca* WSH03-11. They cloned and expressed this GI in *Escherichia coli* BL21, resulting in a recombinant GI with an optimal reaction temperature of 80 °C and half-lives of approximately 2 h at 80 °C and 15 h at 70 °C [10]. Similarly, Neifar et al. derived a GI from *Caldicoprobacter algeriensis*, an organism thriving at an optimal growth temperature of 65 °C, yielding a GI with an optimal reaction temperature of 90 °C [11]. Employing the alternative strategy, Jia et al. immobilized GI from *Thermus oshimai* onto Celite 545, achieving an immobilized GI with an optimal reaction temperature of 85 °C, retaining 70.1% of its initial activity after multiple reaction cycles conducted at 90 °C over 72 h [12].

The proper functioning of metalloenzymes, including GI, depends on the cellular maintenance of tight homeostatic regulation of intracellular metal ion levels (Mn^2+^, Zn^2+^, Ni^2+^, Fe^2+/3+^, etc.) to preserve enzyme structure crucial for catalytic activity while ensuring that ions remain at nontoxic levels compatible with cell survival [13]. GI can be categorized into two classes (Class I and Class II) based on amino acid sequence homology [14]. Importantly, GI activation achieved through the addition of Co^2+^ or Mg^2+^, which is independent of GI class, depends on interactions between these ions and specific GI amino acid residues adjacent to metal ion-binding sites [15]. Maintenance of high GI activity for rapid glucose-to-fructose conversion typically requires substantial amounts of Co^2+^ or Mg^2+^, leading to Co contamination of the final fructose-containing product [16]. However, excessive cellular Co^2+^ intake may trigger DNA oxidative damage and can also harm various human tissues, including the heart, lungs, blood, and thyroid [17,18]. To mitigate potential health risks arising from excessive metal ion consumption in processed foods, ion exchange membrane chromatography is currently conducted to remove added Co^2+^ after the completion of GI-catalyzed isomerization, which is a difficult and costly procedure.

The utilization of free GI for catalyzing D-glucose conversion to D-fructose is constrained by factors such as low GI operational stability, challenges in separating the enzyme from the reaction product, and enzyme non-reusability, which have restricted GI use in industrial-level HCFS production [19,20]. To overcome these limitations, free GI is commonly immobilized onto suitable carriers/matrices through three main strategies: carrier binding (via adsorption or covalent bonding), encapsulation, and covalent cross-linking [21,22]. Nonetheless, the encapsulation of an enzyme may disrupt the proper folding of its protein backbone within the active center, leading to significantly decreased catalytic activity as compared to that of the free enzyme. The covalent cross-linking method for enzyme immobilization can significantly enhance enzyme loading but can lead to food safety issues [23,24,25]. Such issues may be avoided through the use of alternative methods based on physical adsorption through the utilization of hydrophobic and ionic interactions, as well as interactions based on van der Waals forces [26,27]. The choice of carrier plays a crucial role in achieving both effective enzyme immobilization and the preservation of enzyme function across various reaction conditions [28,29]. In recent years, nano-immobilized carriers with large surface areas have been developed that are capable of exerting significant quantum effects as compared to traditional immobilized carriers [30,31].

Metal-organic frameworks (MOFs), characterized by their expansive surface areas and customizable pore structures, offer distinct advantages when applied to enzyme catalysis and immobilization [32,33,34]. For example, Liying Zhu et al. immobilized a GI by attaching it to the MOF ZIF-8 and then affixed the resulting material to permeabilized extracellular surfaces of *Bacillus subtilis* overexpressing trehalose synthase, resulting in the creation of a dual-enzyme system. This system not only supported trehalose production but also converted glucose to fructose as a byproduct. Importantly, the removal of glucose via isomerization prevented glucose-induced inhibition of trehalose synthesis, ultimately leading to increased trehalose yield by increasing the activity of the forward cascade reaction [35]. Similarly, Kaushal et al. achieved effective catalysis of hemicellulose from waste walnut shells to generate xylose by immobilizing xylanase onto a MOF substrate, resulting in a xylose conversion rate of 60.57% [36]. 

While technological advancements have been made in the field of GI immobilization onto MOF carriers, research in this field is still predominantly focused on isomerization reactions requiring supplementation with metal ion activators, which must be removed from the final reaction product, which is an expensive and difficult task. However, the subsequent removal of these ions poses a significant challenge. Overcoming this obstacle may entail conducting these reactions without the initial addition of metal ions in future research. Yet, within the realm of MOF-based enzyme immobilization, no studies have yet emerged aiming to diminish or eliminate the necessity for added metal ion activators while supporting isomerization reactions. This includes investigations into reaction systems employing MOF-immobilized GIs.

In this study, MOF-525 coordination with Co^2+^ was harnessed to generate MOF (Co)-525 that served as a carrier for the immobilization of *Serratia marcescens* glucose isomerase (*Sm*GI) used to synthesize MOF (Co)-525-GI. Subsequently, MOF (Co)-525-GI was subjected to UV-adsorption, SEM-EDS, FTIR spectral analyses, and zeta potential analysis. The enzymatic activity and kinetic analyses of free *Sm*GI and immobilized *Sm*GI under various pH and temperature conditions with or without added Co^2+^ were performed. Additionally, MOF (Co)-525-GI storage stability and reusability were assessed to reveal its industrial potential. Remarkably, MOF (Co)-525-GI was able to effectively catalyze glucose-to-fructose isomerization without continuous supplementation with Co^2+^, highlighting its promise as an immobilized GI for use in industrial HFCS production.

## 2. Materials and Methods

### 2.1. Experimental Materials

*Serratia marcescens* was purchased from the China Center of Industrial Culture Collection (CICC No. 21999, Shanghai, China). Glucose, CoCl_2_·6H_2_O, MgSO_4_·7H_2_O, Na_2_SO_3_, NaOH, and HCl were sourced from Beijing Chemical Works (Beijing, China); benzoic acid, zirconium oxychloride octahydrate (ZrOCl_2_∙8H_2_O), and tris(1-chloro-2-propyl) phosphate (TCPP) were obtained from Shanghai Aladdin Biochemical Technology Co., Ltd. (Shanghai, China). *N*,*N′*-dimethylformamide (DMF), dipotassium hydrogen phosphate, potassium dihydrogen phosphate, and disodium hydrogen phosphate were purchased from Sinopharm Chemical Reagent Co., Ltd. (Beijing, China).

### 2.2. Synthesis and Characterization of MOF (Co)-525-GI

#### 2.2.1. Purification and Culture of *Sm*GI

*Serratia marcescens* was activated and inoculated in 1 L of LB medium and cultured at 28 °C with 170 r/min for 24 h. The bacteria in the medium were centrifuged at 5000× *g* for 30 min. The precipitate was washed three times with Tris-HCl (50 mM pH 7.2) buffer. one gram of precipitate was collected and dissolved in 10 mL Tris-HCl (50 mM pH 7.2) buffer. Then, the precipitate solution was broken by an ultrasonic crusher with an ice bath. Then, the solution was centrifuged at 8000× *g* for 10 min to collect the supernatant. The supernatant was heated at 70 °C for 30 min, and centrifuged at 8000× *g* for 20 min with 4 °C. The supernatant was centrifuged at 1500× *g* for 30 min by a 10 kD ultrafiltration tube and the *Sm*GI solution was collected. The *Sm*GI size was determined by SDS-Page, and the protein concentration was determined using an Enhanced BCA Protein Assay Kit (Shanghai Beyotime P0010, Shanghai, China). The final concentration of *Sm*GI was adjusted to 5.28 mg/mL. 

#### 2.2.2. Preparation of MOF (Co)-525

Synthesis of MOF-525 was performed using the method reported by Chang et al. [37]. ZrOCl_2_ · 8H_2_O (105 mg, 40.73 mmol) and benzoic acid (1.35 g, 1.38 mol) were ultrasonically dissolved in 8 mL of DMF, and then the resulting transparent solution was incubated at 80 °C for 2 h. After the solution was allowed to cool to room temperature, TCPP (47 mg, 7.43 mmol) was added to the mixture, and then it was ultrasonicated for 20 min. Thereafter, it was heated at 80 °C for 24 h and then allowed to cool to room temperature, during which a precipitate formed that was subsequently collected by centrifugation at 10,000× *g* at 4 °C for 10 min. The resulting pellet was washed three times with DMF and then dried under a vacuum at 120 °C for 12 h to generate the final MOF-525 preparation.

For MOF (Co)-525 preparation, 60 mg of MOF-525 was dissolved in 300 mL of DMF, and then CoCl_2_∙6H_2_O (300 mg, 4.20 mmol) was dissolved in the abovementioned solution. After reacting at 120 °C for 18 h, the solution was centrifuged at 10,000× *g*, and then the resulting MOF (Co)-525 precipitate was washed with DMF three times and dried under a vacuum at 120 °C for 12 h to generate the final MOF (Co)-525 preparation.

#### 2.2.3. Characterization of MOF (Co)-525 and MOF (Co)-525-GI

Scanning electron microscopy (SEM) images of MOF (Co)-525 and MOF (Co)-525-GI were obtained using a JSM-6010LA SEM system (CITACHI, Tokyo, Japan). X-ray diffraction (XRD) was carried out using Cu-Ka radiation (α = 1.5418 Å) over a 2θ range of 5–25 at a rate of 1/min using an Empyrean diffractometer (Malvern PANalytical B.V., Almelo, The Netherlands). Fourier-transform infrared (FTIR) spectra of samples prepared using the KBr pellet method were obtained using an FTIR spectrophotometer (Bruker, Ettlingen, Germany), and then data spanning the wavelength range of 4000 cm^−1^ to 500 cm^−1^ were collected and analyzed. UV-Vis absorption spectra were obtained using a SHIMADZU UV-2700 spectrophotometer (UV-2700 Shimadzu, Tokyo, Japan). Zeta potential analysis was performed using a particle size analyzer (Malvern MS 3000, Bristol, UK). Co^2+^ concentrations in reaction solutions were measured using an ICE 3500 atomic absorption spectrometer (Thermo Scientific, Waltham, MA, USA).

### 2.3. Analysis of Enzymatic Activity of MOF (Co)-525-GI

#### 2.3.1. Determination of *Sm*GI Loading Rate

For the determination of the MOF (Co)-525-GI loading rate, 30 mg of dried MOF (Co)-525 was dissolved in 4 mL of Tris-HCl (50 mM pH 7.2) buffer solution. After sonication for 30 min, 1 mL of 5.28 mg/mL *Sm*GI extracted from *S. marcescens* was added to the MOF (Co)-525 solution to generate a 5 mL reaction system that was stirred for 2 h at 4 °C. During stirring, a precipitate formed that was collected via centrifugation at 5000× *g* for 15 min. After centrifugation, the *Sm*GI concentration in the supernatant was determined (*X*_1_). The precipitate was washed with Tris-HCl(50 mM pH 7.2) buffer three times, yielding *Sm*GI concentrations in subsequent washes designated as *X*_2_, *X*_3_, and *X*_4_, while the *Sm*GI concentration before its addition to the MOF (Co)-525 solution was designated as *X*. *Sm*GI concentrations were determined using an Enhanced BCA Protein Assay Kit (Shanghai Beyotime P0010, Shanghai, China). The *Sm*GI load rate (*Y*) was calculated using the following formula:

$$Y=\frac{X-X_{1}-X_{2}-X_{3}-X_{4}}{X}*100\%$$

#### 2.3.2. Effects of pH and Temperature on Immobilized and Free *Sm*GI Enzymatic Activities

The effect of pH on the enzymatic activity of free *Sm*GI was measured after 0.3 mL of a solution of free *Sm*GI was added to a 2.7 mL volume of 50% glucose solutions of pH 3.5, 4.5, 5.5, 6.5, 7.5, 8.5, or 9.5. The effect of pH on enzymatic activities of either MOF-525-GI or MOF (Co)-525-GI in a 3 mL reaction system was assessed after 20 mg of these reaction systems were added to 3 mL of 50% glucose solutions of pH 3.5, 4.5, 5.5, 6.5, 7.5, 8.5, or 9.5. All reactions were allowed to proceed at 60 °C for 1 h, and then the immobilized enzyme was removed from reaction supernatants by passing them through 0.22-μm membrane filters. Supernatant fructose concentrations were measured using a Fructose Assay Kit (Nanjing Jiancheng A085-1-1, Nanjing, China).

To assess the temperature optimum for free *Sm*GI, MOF-525-GI, and MOF (Co)-525-GI, 0.3 mL of free *Sm*GI was added to 2.7 mL of a 50% glucose solution at pH 7.5, while 20 mg of MOF (Co)-525-GI was added to 3 mL of a 50% glucose solution at pH 7.5. After the three reaction systems were incubated for 1 h at 30, 40, 50, 60, 70, 80, or 90 °C, the immobilized enzyme was removed from reaction supernatants by passing it through 0.22 μm membrane filters, and then fructose concentrations in filtrates were measured as described above.

#### 2.3.3. Determination of Biochemical and Operation Properties

The enzymatic activities of free and immobilized *Sm*GI were assessed in 3 mL reaction volumes based on enzyme catalytic activity for the conversion of glucose to fructose. For the assessment of free or immobilized *Sm*GI catalytic activity, 0.3 mL of a solution containing 5.28 mg/mL free *Sm*GI was added to 2.7 mL of deoxygenated solutions containing various concentrations of glucose substrate (2.5, 5, 10, 20, 40, 60, 80, 100, 200, and 400 mM) to generate 3 mL reaction systems, while 20 mg of MOF (Co)-525-GI was added to 3 mL volumes of the same glucose solutions. After incubating each reaction at 60 °C for 1 h, immobilized *Sm*GI was collected using centrifugation for later use. Fructose concentrations of all supernatants were immediately measured after passing supernatants through 0.22 μm membrane filters. *K*_m_ and *V*_max_ values of reactions containing free and immobilized *Sm*GI were calculated using the Lineweaver–Burk equation:

$$\frac{1}{V_{0}}=\frac{K_{m}}{V_{max}*[S]}+\frac{1}{\left[S\right]}$$

where V_0_ is the reaction rate (μM min^−1^), *V*_max_ is the maximum reaction rate (μM min^−1^), [S] is the glucose concentration (mM mL^−1^), and *K*_m_ is the Michaelis–Menten constant.

#### 2.3.4. Assessments of Reusability and Storage Stability of MOF (Co)-525-GI

To assess MOF (Co)-525-GI reusability and storage stability, 20 mg of MOF (Co)-525-GI was added to 3 mL of a 50% (*w*/*v*) glucose solution at pH 7.5. After the reaction was allowed to proceed at 70 °C for 1 h, the immobilized enzyme was collected using centrifugation for later use. The fructose concentration in the supernatant was measured after supernatants were passed through 0.22 μm membrane filters, as described in Section 2.3.3. The relative enzymatic activities of free *Sm*GI and MOF (Co)-525-GI stored at 4 °C were determined every 15 days during storage and compared to respective free *Sm*GI and MOF (Co)-525-GI catalytic activities prior to storage (which were set to 100%). 

### 2.4. Statistical Analysis

Statistical analyses were performed using GraphPad Prism 10.1 software (GraphPad Software Inc., San Diego, CA, USA) based on calculated mean and standard error values obtained for three experimental replicates. Graphs were analyzed using Origin 2022 (Origin 2022, OriginLab, Northampton, MA, USA).

## 3. Results and Discussion

### 3.1. Characterization of MOF (Co)-525-GI Structural Features and Elemental Composition

#### 3.1.1. SDS-Page of *Sm*GI and SEM-EDS Analysis of MOF (Co)-525-GI

**Figure 1:**
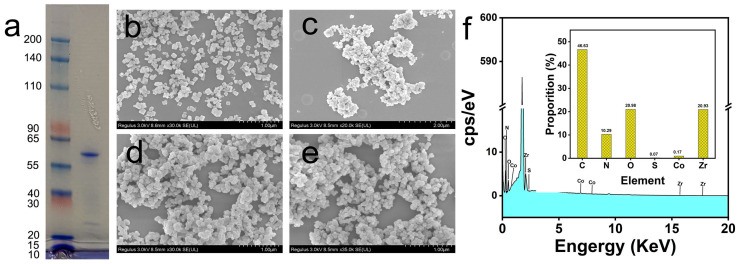
*Sm*GI SDS-Page and the SEM-EDS analysis of MOF(Co)-525-GI. (**a**) *Sm*GI SDS-Page; (**b**) SEM image of MOF-525; (**c**) SEM image of MOF-525-GI; (**d**) SEM image of MOF(Co)-525; (**e**) SEM image of MOF(Co)-525-GI; (**f**) EDS spectrum of MOF (Co)-525-GI.

The protein bands of *Sm*GI were observed in 10% acrylamide gel. SDS-PAGE results of *Sm*GI used in the experiment are shown in Figure 1a. The main bind at approximately 60 kD was detected, which is consistent with published reports [38]. Also, the purity of the protein bind showed that the protein was suitable for further experiments. The immobilization rate of MOF (Co)-525 for *Sm*GI was 43.2% following the formula in Section 2.3.1, which was higher than previously reported [39]. Since the immobilization rate was 43.2%, with the description in Section 2.3.1, 30 mg of MOF (Co)-525 was immobilized in 2.28 mg of *Sm*GI, and the *Sm*GI content of MOF (Co)-525 was 76.03 mg/g.

SEM analyses of MOF-525 and MOF (Co)-525 structural features revealed uniform particles with diameters of approximately 100 nm with regular cubic shapes (Figure 1b,d), which is consistent with corresponding features reported for MOF-525 synthesized by Ting-Hsiang Chang et al., thereby confirming successful MOF-525 synthesis [37]. After the immobilization of *Sm*GI onto MOF-525 or MOF (Co)-525 surfaces, MOF morphologies exhibited cubic structures resembling those observed for MOF-525 or MOF (Co)-525 prior to *Sm*GI immobilization (Figure 1c,e). 

EDS analysis was conducted to determine the elemental composition of MOF (Co)-525 (Figure 1f). EDS results demonstrated the presence of C, O, S, Co, and Zr in MOF (Co)-525-GI at mass ratios of 46.63%, 10.29%, 20.98%, 0.07%, 0.17%, and 20.93%, respectively. With excess Co^2+^ removed from the reaction of MOF-525, the Co^2+^ signal showed that Co^2+^ successfully coordinated within MOF (Co)-525 (Figure 1f). Sulfur, an element found in proteins but not in MOF (Co)-525, was detected in the EDS spectrum, thus indicating that *Sm*GI was successfully immobilized onto the MOF (Co)-525 substrate.

#### 3.1.2. Results of XRD, UV Absorption Spectroscopy, FTIR Spectroscopy, and Zeta Potential Analyses

**Figure 2:**
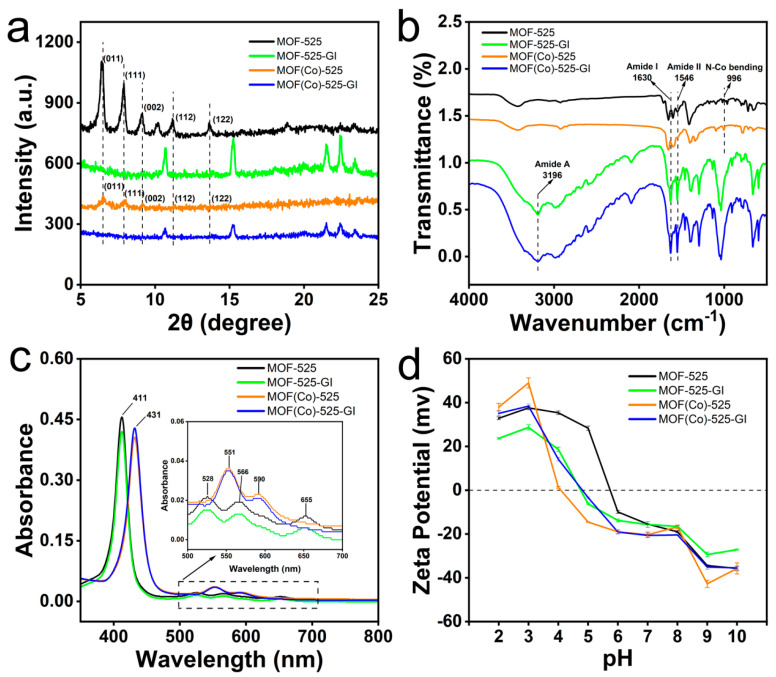
Characterization of MOF-525-GI. (**a**) XRD crystal structure of MOF-525 (Black), MOF-525-GI (Green), MOF(Co)-525 (Orange), MOF(Co)-525-GI (Blue); (**b**) Infrared absorption spectra of MOF-525 (Black), MOF-525-GI (Green), MOF(Co)-525 (Orange), MOF(Co)-525-GI (Blue); (**c**) UV absorption spectra of MOF-525 (Black), MOF-525-GI (Green), MOF(Co)-525 (Orange), MOF(Co)-525-GI (Blue); (**d**) Zeta potential of MOF-525 (Black), MOF-525-GI (Green), MOF(Co)-525 (Orange), MOF(Co)-525-GI (Blue) at pH 2−10.

Results of XRD analysis of the crystal structure of dried MOF-525 revealed main characteristic peaks located at 2θ = 6.5°, 7.9°, 9.2°, 11.1°, 13.6°, and 13.9° corresponding to (011), (111), (002), (112), (122), and (525) MOF-525 planes (Figure 2a), aligning with previously reported results [40]. Moreover, the MOF-525 powder X-ray diffraction (PXRD) pattern exhibited a high sharp peak profile, indicating the high purity of synthesized MOF-525 crystals. Notably, similar XRD results were obtained for MOF (Co)-525 and MOF-525, indicating that the presence of Co^2+^ in the reaction system did not alter the MOF-525 structure. In contrast, immobilized *Sm*GI did not produce clear sharp XRD peaks, indicating that *Sm*GI was affixed to MOF (Co)-525 surfaces.

FTIR results obtained for MOF (Co)-525 and MOF (Co)-525-GI within the wavelength range of 4000–500 cm^−1^ were consistent with spectra reported by Chang et al., thus confirming the successful synthesis of MOF-525 [37]. Comparisons of FTIR spectra obtained for MOF-525 and MOF (Co)-525 revealed that added Co^2+^ coordinated with MOF-525, as indicated by the appearance of a new peak at 996 cm^−1^ [41]. 

After *Sm*GI was immobilized on MOF-525 or MOF (Co)-525, new FTIR spectral peaks corresponding to amide A, amide I, and amide II bonds appeared (Figure 2b). According to published results, the amide A band is mainly attributed to an N-H stretching vibration in resonance with amide II, while the amide I absorption band corresponds primarily to the amide group C=O stretching vibration, and the amide II absorption band corresponds to N-H bending and C–N stretching vibrations [42]. Notably, an analysis of *Sm*GI spectral bands within amide A, amide I, and amide II regions of free *Sm*GI and MOF (Co)-525-GI revealed that the structure of *Sm*GI remained intact after immobilization. 

Changes in porphyrin UV absorption spectral Soret and Q bands are important indicators of the metallization status of the porphyrin center. As shown in Figure 2c, characteristic UV absorption peak results revealed a maximum MOF-525 absorption peak near 411 nm within the Soret band region and three additional peaks at 528, 566, and 655 nm. The peak at 411 nm was shifted to 431 nm in MOF (Co)-525 due to the coordination of Co with MOF-525, as consistent with published results indicating successful synthesis of Co-TCPP [41,43]. Moreover, peaks at 528 nm, 566, and 655 were replaced by peaks at 551 nm and 590 nm, while *Sm*GI UV absorbance spectra did not change after *Sm*GI immobilization.

Zeta potential is an important indicator of colloidal dispersion system stability [44,45]. Here, zeta potential measurements revealed the greatest MOF (Co)-525 stability at pH = 3 or pH = 9. At a pH of >5, both MOF-525 and MOF (Co)-525 in the buffer carry negative charges (Figure 2d). In contrast, zeta potentials of MOF-525(Co)-GI obtained under different pH conditions clearly indicate that the material in solution is extremely unstable within the pH range of 4–6 and relatively stable within the pH range of 6–8. Within the latter pH range, good dispersion of MOF (Co)-525-GI enhances its ability to effectively interact with the substrate, thereby increasing its catalytic activity.

### 3.2. Effects of Temperature and pH on SmGI Enzymatic Activity

**Figure 3:**
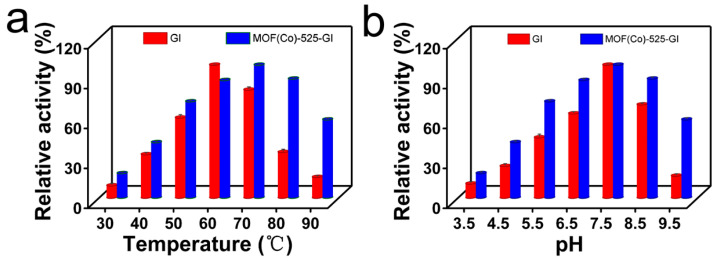
Effect of temperature and pH for free *Sm*GI and MOF (Co)-525-GI enzyme activity. (**a**) The effect of temperature on the activity of free *Sm*GI (Red) and MOF (Co)-525-GI (Blue) from 30 °C to 90 °C; (**b**) the effect of pH on the activity of free *Sm*GI (Red) and MOF (Co)-525-GI (Blue) at pH 3.5–9.5.

Environmental factors such as pH and temperature greatly influence enzyme activity, with excellent stability, serving as an important indicator of immobilized enzyme industrial potential [46]. Temperature can provide energy for the isomerization *Sm*GI-catalyzed conversion of glucose to fructose. Based on this premise, we investigated the effect of temperature on free and immobilized *Sm*GI catalytic activities. Our experimental results demonstrated good enzymatic activity of immobilized *Sm*GI at temperatures between 50 and 80 °C, while good enzymatic activity of free *Sm*GI was observed only at 60 °C and optimal MOF (Co)-525-GI enzyme activity was observed only at 70 °C, as shown in Figure 3a. 

As compared to the optimal temperature of free *Sm*GI, MOF (Co)-525-GI exhibited optimal catalytic activity at a temperature approximately 10 °C higher (Figure 3a). During the process of *Sm*GI conversion of glucose to fructose, a thermodynamic equilibrium exists between glucose and fructose conversion reactions after glucose conversion reaches a certain level [47,48]. Notably, with increasing temperature, the conversion of glucose to fructose becomes more favorable and therefore would be better supported by (Co)-525-GI than by free *Sm*GI, highlighting the enhanced suitability of MOF (Co)-525-GI for high-temperature industrial food processing.

We also assessed MOF (Co)-525-GI stability under different pH conditions within the pH range of 3.5–9.5 (Figure 3b) and observed the highest enzyme activity at pH 7.5 and greatest stability within the pH range of pH 5.5–8.5. However, when the pH was >9, the activity of the immobilized enzyme decreased drastically, due to potential destabilization of the *Sm*GI conformation and possible decomposition of the MOF-525 nanometal-organic framework under alkaline conditions [49,50]. Importantly, the pH of the reaction system tended to decrease with the progression of the *Sm*GI catalytic isomerization reaction. Nevertheless, the relatively greater stability of MOF (Co)-525-GI under weakly acidic conditions as compared to that of free *Sm*GI underscores the greater suitability of MOF (Co)-525-GI for industrial HFCS production.

### 3.3. Kinetic Studies of MOF (Co)-525-GI Activity

**Figure 4:**
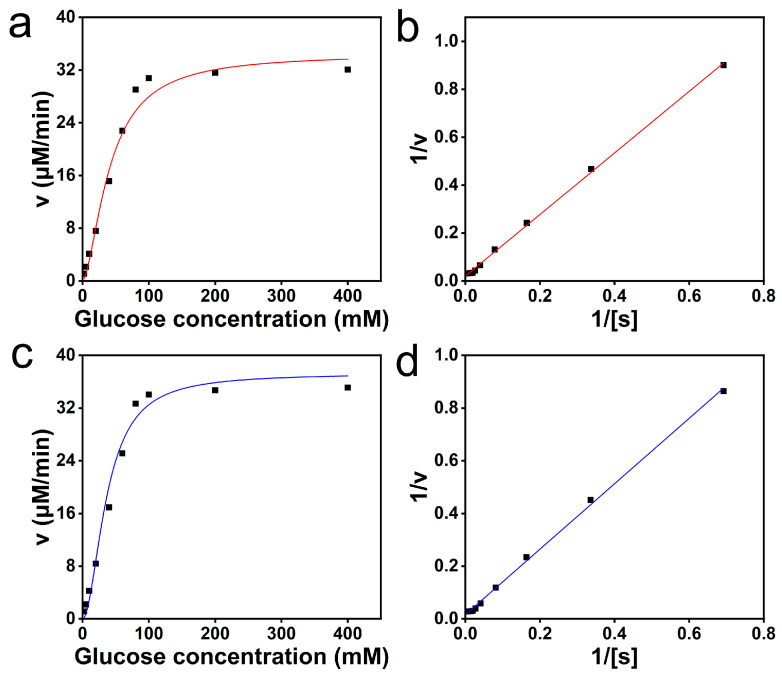
Influence of substrate concentration on the activity of immobilized enzyme in a reaction system without Co^2+^ addition. (**a**) Activity of free *Sm*GI with substrate concentrations ranging from 2.5–400 mM; (**b**) Linewever–Burk double reciprocal plot of free *Sm*GI activity at substrate concentrations of 2.5–400 mM; (**c**) activity of MOF (Co)-525-GI with substrate concentrations ranging from 2.5–400 mM; (**d**) Linewever–Burk double reciprocal plot of MOF (Co)-525-GI activity at substrate concentrations of 2.5–400 mM.

During the MOF (Co)-525-GI-catalyzed reaction, enzyme activity was detectable over the substrate concentration range of 2.5–400 mM (Figure 4a,c) and a good linear relationship was observed between glucose isomerization rate and concentration at low glucose concentrations. However, as the glucose concentration increased, the reaction rate gradually reached a maximum (Figure 4a,c), as indicated by Lineweaver–Burk double reciprocal analysis results (Figure 4b,d) and the maximum reaction speed (*V*_max_) and *K*_m_ of *Sm*GI (Table 1). A comparison of *K*_m_ values obtained for *Sm*GI and MOF (Co)-525-GI revealed a greater value for MOF (Co)-525-GI, indicating reduced enzyme-substrate affinity and associated reduced enzymatic activity due to decreased enzyme dispersion post-immobilization [48]. Notably, the substrate affinity of MOF (Co)-525-GI in our experiments was greater than that reported in the literature, highlighting its promise for use in industrial food processing operations [12].

Importantly, the addition of Co^2+^ increased the enzymatic activity of free *Sm*GI (Table 1), an effect that may have been due to Co^2+^ induction of increased *Sm*GI affinity for the substrate. In contrast, the effect of added Co^2+^ on the enzymatic activity of MOF (Co)-525-GI was less pronounced than Co^2+^ enhancement of free *Sm*GI activity, whereby in the absence of added Co^2+^ (low Co^2+^ concentration), MOF (Co)-525-GI exhibited optimal enzyme activity that would render it particularly suitable for use in food-processing applications. Moreover, in the absence of added Co^2+^, the catalytic activity of MOF (Co)-525-GI was higher than that of MOF-525-GI, due to *Sm*GI activation by Co^2+^ within the MOF (Co)-525-GI porphyrin center [51]. Collectively, these results demonstrate that MOF (Co)-525-GI possessed good long-term substrate affinity and stable enzyme activity in the absence of added metal ion activator, providing a theoretical basis for reducing the use of metal ion activators such as CoCl_2_ in the HFCS production process.

### 3.4. Assessments of MOF (Co)-525-GI Reusability and Stability

**Figure 5:**
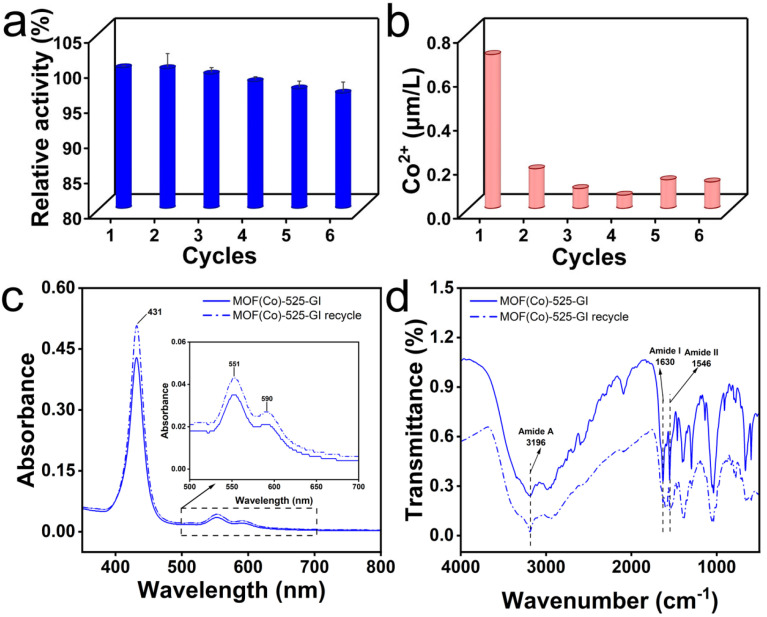
The reusability of the MOF (Co)-525-GI. (**a**) The MOF (Co)-525-GI enzyme activity after six cycles of use. (**b**) The concentration of Co^2+^ in the reaction system after six cycles of use of MOF (Co)-525-GI. (**c**) The UV spectra of MOF (Co)-525-GI before (Line) and after six cycles using (Dot-line). (**d**) The FTIR spectra of MOF (Co)-525-GI before (Line) and after six cycles using (Dot-line).

Immobilized *Sm*GI was assessed for reusability, with the results demonstrating that MOF (Co)-525-GI retained 96.38% of its initial activity after six reaction cycles (Figure 5a). Moreover, the structure of the immobilized enzyme remained intact during cycling, as indicated by unchanging UV spectra during cycling (Figure 5c) and unchanging FTIR spectra indicating complete retention of Co^2+^ during cycling (Figure 5d). These results demonstrate that immobilized *Sm*GI retained greater enzyme activity over six reaction cycles than that retained by free *Sm*GI, thereby enhancing the industrial applicability of *Sm*GI for food processing.

Atomic absorption spectrophotometric measurements of Co^2+^ concentration in the MOF (Co)-525-GI reaction system over six reaction cycles (Figure 5b) indicated that during cycling, the Co^2+^ concentration remained essentially constant and was approximately one-thousandth that of the free *Sm*GI reaction system with continuous Co^2+^ supplementation. Therefore, coordination of Co^2+^ at the MOF (Co)-525-GI porphyrin center plays an important role in *Sm*GI enzyme activity by reducing added Co^2+^ to maintain enzyme activity over repeated reaction cycles. These results clarified that the construction of a reaction system for HFCS production with low Co^2+^ addition is possible. 

**Figure 6:**
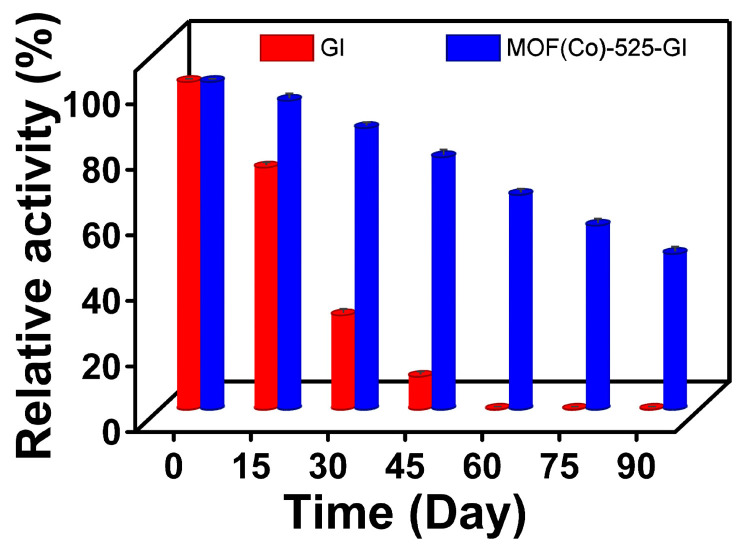
The storage stability of MOF (Co)-525-GI. The relative activity of free *Sm*GI (Red) and MOF (Co)-525-GI (Blue) in 90 days of storage.

An analysis of storage stabilities of free *Sm*GI and MOF (Co)-525-GI based on enzymatic activities measured every 15 days revealed that *Sm*GI stored at 4 °C retained 73.79% of its initial enzymatic activity after 15 days, 28.54% of its initial activity after one month, and no activity after 60 days (Figure 6). In contrast, after 30 days of storage, MOF (Co)-525-GI enzymatic activity remained higher (85.92%) than that reported for *Sm*GI immobilized onto the upper critical solution temperature (UCST)-responsive polymers carrier [48]. Moreover, on the 15th day of storage, MOF (Co)-525-GI residual activity was significantly higher than that of free *Sm*GI and remained high (47.4%) after 90 days of storage. Therefore, immobilization endowed *Sm*GI with significantly greater long-term storage stability compared to that of free *Sm*GI, further highlighting MOF (Co)-525-GI suitability for food-processing applications.

## 4. Conclusions

In this study, we successfully synthesized MOF (Co)-525-GI by immobilizing *Sm*GI onto the carrier MOF (Co)-525. Our comprehensive analysis of MOF (Co)-525-GI physical and enzymatic characteristics revealed that *Sm*GI retained its crystalline structure after immobilization and exhibited high enzymatic activity even in the absence of added Co^2+^. Notably, the immobilized enzyme displayed an optimal reaction temperature of 70 °C, surpassing the free *Sm*GI optimal temperature of 60 °C. This result suggests that the immobilized enzyme could function outside of the conventional GI enzymatic kinetic equilibrium, resulting in enhanced yield of the reaction product fructose. Also, without extra Co^2+^ iron added, the MOF (Co)-525-GI enzyme activity was similar to the free Co ion addition of immobilized *Sm*GI. But the MOF (Co)-525-GI reduced the Co ion adding in the enzyme reaction system. Our findings also demonstrated broader pH adaptability and superior storage stability of MOF (Co)-525-GI than that observed for free *Sm*GI. Remarkably, MOF (Co)-525-GI retained 47.4% of its initial activity after 90 days of storage. This study introduces a promising approach to *Sm*GI immobilization, laying a theoretical foundation to reduce the reliance on metal ions in HFCS production. Such a strategy not only holds the potential to enhance food safety but also streamlines the conversion process by eliminating the need to remove Co^2+^, thereby reducing production costs.

## Figures and Tables

**Table 1:** Kinetic behavior of free GI and immobilized enzymes.

	*V*_max_ (μM/min)	*K*_m_ (mM)	*K*_cat_ (S^−1^)
*Sm*GI	34.46 ± 2.00	44.27 ± 3.10	68.53 ± 3.98
*Sm*GI + Co^2+^	52.21 ± 1.86	28.67 ± 1.23	103.83 ± 3.70
MOF-525-GI	23.11 ± 1.84	56.32 ± 5.43	46.53 ± 3.71
MOF-525-GI + Co^2+^	38.85 ± 1.71	52.58 ± 2.45	78.22 ± 3.45
MOF(Co)-525-GI	37.24 ± 1.91	46.25 ± 3.03	90.2 ± 4.63

## Data Availability

The original contributions presented in the study are included in the article/Supplementary Materials, further inquiries can be directed to the corresponding authors.

## Supplementary Materials

The following are available online at https://www.mdpi.com/article/10.3390/foods13040527/s1, Figure S1: SmGI SDS-Page.
