# Supplementary material for: Enhanced High-Fructose Corn Syrup Production: Immobilizing Serratia marcescens Glucose Isomerase on MOF (Co)-525 Reduces Co2+ Dependency in Glucose Isomerization to Fructose
Source: Foods. 2024 Feb 8;13(4):527. doi: 10.3390/foods13040527 (PMC10888103; doi:10.3390/foods13040527)
Supplement: Supplementary file 1 [file foods-13-00527-s001.zip › foods-2846444-supplementary.pdf]

Supporting Information

1. Purification and Culture of SmGI

Serratia marcescens was activated and inoculated in 1 L liquid LB medium and cultured at 28 °C and 170 r/min for 24 h. The bacteria in the medium were centrifuged in a high-speed refrigerated centrifuge at 5000 g centrifugal force for 30 min. The collected bacteria are stored in -20 °C refrigerator for use. Weigh the bacteria and place the wet bacteria in the Tris-HCl buffer at the ratio of 1 g/10 mL Tris-HCl. Under the condition of ice bath, the bacteria were broken by ultrasonic crusher. The heat-resistant miscellaneous protein was removed in a water bath at 60 °C for 30 min, followed by centrifuge at -4 °C with a high speed refrigerated centrifuge at 8000 g centrifugal force for 20 min to remove the precipitation, and the resulting supernatant was filtered by 10000M ultrafiltration tube to remove small molecular miscellaneous protein to obtain SmGI, which was stored in a refrigerator at 4 °C. The SmGI size was determined by SDS-Page, and the protein concentration was about 5.28 mg/mL (Figure S1).

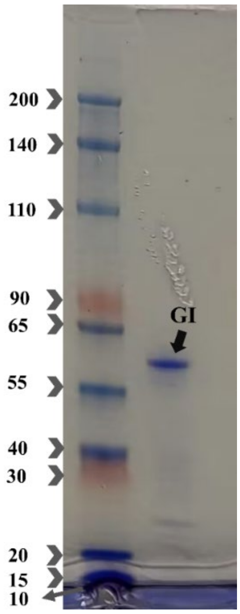

Figure S1. SmGI SDS-Page
